# Multi-center evaluation of baseline neutrophil-to-lymphocyte (NLR) ratio as an independent predictor of mortality and clinical risk stratifier in idiopathic pulmonary fibrosis

**DOI:** 10.1016/j.eclinm.2022.101758

**Published:** 2022-12-01

**Authors:** Theresia A. Mikolasch, Peter M. George, Jagdeep Sahota, Thomas Nancarrow, Shaney L. Barratt, Felix A. Woodhead, Vasilis Kouranos, Victoria S.A. Cope, Andrew W. Creamer, Silan Fidan, Balaji Ganeshan, Luke Hoy, John A. Mackintosh, Robert Shortman, Anna Duckworth, Janet Fallon, Helen Garthwaite, Melissa Heightman, Huzaifa I. Adamali, Sarah Lines, Thida Win, Rebecca Wollerton, Elisabetta A. Renzoni, Matthew Steward, Athol U. Wells, Michael Gibbons, Ashley M. Groves, Bibek Gooptu, Chris J. Scotton, Joanna C. Porter

**Affiliations:** a CITR, UCL Respiratory, UCL, London, UK; b Interstitial Lung Disease Service, UCLH NHS Trust, London, UK; c Interstitial Lung Disease Unit, Royal Brompton Hospital, UK; d National Heart and Lung Institute, Imperial College London, UK; e College of Medicine & Health, University of Exeter, Exeter, UK; f Academic Department of Respiratory Medicine, Royal Devon & Exeter NHS Foundation Trust, Exeter, UK; g Bristol Interstitial Lung Disease Service, North Bristol NHS Trust, Bristol, UK; h Academic Respiratory Unit, University of Bristol, Bristol, UK; i Institute for Lung Health and Leicester Interstitial Lung Disease Service and NIHR Leicester Biomedical Research Centre - Respiratory, Glenfield Hospital, Groby Road, Leicester, LE3, UK; j Department of Respiratory Sciences and Leicester Institute of Structural & Chemical Biology University of Leicester, Henry Wellcome Building, Lancaster Road, Leicester, LE1 5HB, UK; k Institute of Nuclear Medicine, UCL and Department of Nuclear Medicine UCLH, UK; l The Prince Charles Hospital, Queensland, Australia; m Department of Respiratory Medicine, Somerset Lung Centre, Musgrove Park Hospital, Taunton, UK; n Lister Hospital, North East Herts Trust, Stevenage UK

**Keywords:** Biomarker, Leukocyte, Mortality, IPF, Interstitial Lung Disease, ILD

## Abstract

**Background:**

Idiopathic pulmonary fibrosis (IPF) is a progressive, fatal disorder with a variable disease trajectory. The aim of this study was to assess the potential of neutrophil-to-lymphocyte ratio (NLR) to predict outcomes in IPF.

**Methods:**

We adopted a two-stage discovery (n = 71) and validation (n = 134) design using patients from the UCL partners (UCLp) cohort. We then combined discovery and validation cohorts and included an additional 794 people with IPF, using real-life data from 5 other UK centers, to give a combined cohort of 999 patients. Data were collected from patients presenting over a 13-year period (2006–2019) with mean follow up of 3.7 years (censoring: 2018–2020).

**Findings:**

In the discovery analysis, we showed that high values of NLR (>/ = 2.9 vs < 2.9) were associated with increased risk of mortality in IPF (HR 2.04, 95% CI 1.09–3.81, n = 71, p = 0.025). This was confirmed in the validation (HR 1.91, 95% CI 1.15–3.18, n = 134, p = 0.0114) and combined cohorts (HR 1.65, n = 999, 95% CI 1.39–1.95; p < 0·0001). NLR correlated with GAP stage and GAP index (p < 0.0001). Stratifying patients by NLR category (low/high) showed significant differences in survival for GAP stage 2 (p < 0.0001), however not for GAP stage 1 or 3. In a multivariate analysis, a high NLR was an independent predictor of mortality/progression after adjustment for individual GAP components and steroid/anti-fibrotic use (p < 0·03). Furthermore, incorporation of baseline NLR in a modified GAP-stage/index, GAP–index/stage-plus, refined prognostic ability as measured by concordance (C)-index.

**Interpretation:**

We have identified NLR as a widely available test that significantly correlates with lung function, can predict outcomes in IPF and refines cohort staging with GAP. NLR may allow timely prioritisation of at-risk patients, even in the absence of lung function.

**Funding:**

Breathing Matters, 10.13039/100004330GSK, CF Trust, BLF-Asthma, 10.13039/501100000265MRC, NIHR Alpha-1 Foundation.

**Research in context**

**Evidence before this study**

There is an urgent need for biomarkers to better stratify patients with idiopathic pulmonary fibrosis (IPF) for clinical trials and transplant allocation. We investigated whether the neutrophil-to-lymphocyte ratio (NLR) in the peripheral blood could refine the current clinical scoring system (GAP: gender, age, and physiology) to identify cohorts of patients with IPF at higher risk of poor outcomes. We searched the scientific literature using PubMed to identify studies in which the baseline NLR had been used to predict outcomes for patients with IPF. We used the search terms “IPF”, “pulmonary fibrosis” and “NLR” and did not use language or date restrictions. We identified seven studies that specifically considered NLR as a biomarker in IPF: Of these two were small single centre studies and a third study measured NLR in bronchoalveolar lavage (BAL). Nathan et al. included 1334 patients with IPF from ASCEND (Study 016; NCT01366209) and CAPACITY (Studies 004 and 006; NCT00287716 and NCT00287729) as a discovery cohort and placebo-treated IPF patients from two independent Phase III, trials of IFN-γ-1b (GIPF-001 (NCT00047645) and GIPF-007 (NCT00075998) as a validation cohort. Finally, the most recent study compared the predictive potential of NLR in fibrosing hypersensitivity pneumonitis (fHP) compared with IPF. None of these studies validated the ability of NLR to predict mortality beyond 12 months in IPF and there is no data on the incorporation of NLR in an adjusted GAP score. A simple, cheap, widely available, circulating biomarker that refines GAP score at presentation would add substantially to IPF care.

**Added value of this study**

In this study, we identified two groups of patients with IPF based on NLR at diagnosis. Those with high NLR (>/ = 2.9) had significantly higher mortality than those with low NLR (<2.9; HR 2.04; 95% CI 1.09–3.81; p = 0.025). We validated our findings using real-life data collected from 928 patients with IPF from 6 different UK centres. The incorporation of baseline NLR in a modified GAP-stage/index, (GAP/index)-plus further refined cohorts for prognostic prediction of this clinical scoring system.

**Implications of all the available evidence**

We have shown that the NLR, which is calculated from full blood count with differential, is an inexpensive, easy to obtain, widely available, reproducible, and independent prognostic biomarker in patients with IPF. NLR can significantly refine the predictive ability of GAP index at diagnosis with an NLR of >/ = 2.9 identifying patients at increased risk of deterioration that require more rapid assessment in specialist centres. In addition, we have shown that NLR significantly correlates with lung function (FVC and DLco) and may be particularly helpful in situations where lung function cannot be performed by the patient, or is not easily available, such as in remote areas and during a pandemic. Further evaluation of the utility of NLR measurement for therapeutic decision making in IPF is warranted.

## Introduction

Idiopathic pulmonary fibrosis (IPF) is a progressive, fatal disorder with a very variable disease trajectory. Available treatments for IPF are expensive and merely slow disease progression with frequent side-effects. A prognostic biomarker would guide treatment decisions, timing of lung transplant or end of life care and help patients and clinicians to plan.

Clinical cohort staging in IPF relies on the Gender, Age, Physiology (GAP) index (a score from 0 to 7) with associated GAP stage (I-III), a static measure unable to identify rapidly deteriorating patients, or assess treatment response. There is an unmet need for biomarkers to guide a personalized approach to care, as well as for cohort stratification in clinical trials. Only two biomarkers have been validated to refine the GAP staging system by identifying high and low risk patients within a given GAP stage. The first used a 52-gene expression signature, an approach that requires calibration against a control cohort,^1^ and the second measured glucose uptake in the lung with Positron Emission Tomography (PET).^2^ Both biomarkers require specialist expertise and are costly, limiting their practicality. The ideal biomarker would be measurable in the blood using a simple and widely available test and would predict prognosis and potentially response to therapy.

The Neutrophil-Lymphocyte Ratio (NLR) is easily and inexpensively measured from a complete blood count (CBC), and has indicated severity in studies of diabetes,^3^^,^^4^ cardiovascular^5^ and renal disease,^6^ COPD,^3^ malignancy^7^ and COVID-19.^8^^,^^9^ NLR can also predict development and severity of lung fibrosis in patients with systemic sclerosis,^10^ dermatomyositis/polymyositis^11^ and a composite endpoint of ‘absolute decline in 6MWD ≥50 m or death’ at 12 months in IPF.^12^ It is not known whether addition of NLR to GAP will refine clinical cohort staging in IPF and guide management.

Here we use a two-stage derivation and validation model to determine a discriminatory cut-off for low (<2.9) or high (>/ = 2.9) NLR. We combined our derivation and validation cohorts with additional external cohorts to give a combined cohort of 999 patients to then investigate the ability of NLR to refine the prognostic power of the clinical GAP score in IPF.

## Methods

### Study design and participants

An observational study to evaluate NLR as an independent mortality risk predictor in IPF. Derivation cohort, 71 IPF patients enrolled at UCLH (2008–18). Additional cohort of 928 patients comprising: Validation cohort for NLR of 134 patients from UCLH (2006–18); External cohorts of 794: 279 IPF patients from Royal Brompton and Harefield NHS Trust (RBH) (2006–2018); cohort of 515 IPF patients from Southwest and Leicester (SW&L): Royal Devon and Exeter (RDE) Hospital (300), North Bristol (NB) NHS Trust (85), Taunton and Somerset (TS) NHS Foundation Trust (30); and University Hospitals of Leicester (UHL; 90), (2011–2019). Total combined cohort, N = 999. Inclusion criteria: diagnosis of IPF; baseline pulmonary function tests and CBC. Exclusions: malignancy or haematological disorder; infection at time of CBC (CRP >/ = 20 mg/L, clinical/imaging signs of infection); cytotoxic drugs. Exclusion from derivation cohort if on prednisolone >/5 mg or equivalent at time of CBC, or if insufficient data. CBC was taken at time of IPF diagnosis and analysed according to local NHS protocols. There was no standardization of analysis, or of normal ranges, between sites. An inclusion level of CRP <20 mg/L was chosen as shown to be discriminatory for excluding bacterial infections in adults^13^

Antifibrotic data was available for the Southwest cohorts (RD&E, MPH, NBT) and RBH for patients on antifibrotics for >6 weeks. However, neither time nor duration of therapy, was recorded.

We have previously reported part of our UCLH internal derivation and validation cohorts, 208 patients, as an abstract.^14^ The 515 patients from NB/RDE/TS/UHL were reported as part of a larger cohort comparing basic outcome predictors in IPF versus fHP^15^

### Outcomes

Primary outcome measure was transplant free survival from CBC measurement to death (all-causes) or transplant in high and low NLR groups using the following censoring: UCLH 28/6/2018, RBH 30/1/2020, SW&L 12/7/2019. Secondary outcome was assessment of NLR as a mortality predictor in comparison to GAP index-predicted mortality (Table S1)^16^ and independence of GAP index.

### Statistical analysis

A non-biased empirical Cumulative Distribution Function, eCDF plot of baseline NLR of the derivation cohort was used to determine the median NLR. Harrell's concordance (C)-index was used to determine the ability of NLR to predict outcome accurately with increasing time from baseline. Different models are compared by C-index with an increase in the C-index indicating an improvement in the model. Analysis was performed using STATA 15 (Stata Corp, College Station, Texas). Fisher's exact test and unpaired two tailed t-tests were used to calculate significance between different group characteristics. Although a normal distribution of data was not formally proven, histograms of lung function, age, and GAP index between high and low NLR groups, were not observed to be skewed and with no extreme outliers, and, given the large sample size, the application of the t-test was acceptable.^17^ Further sensitivity analysis was performed using non-parametric tests. All p-values are reported for two-sided confidence intervals. A p-value of <0.05 was considered significant. However, as C-indices are not sensitive enough to detect statistical differences between models, p values for differences in C-indices are not reported.

#### Survival analysis

Both transplant and death were events. Univariate analysis was used to calculate risk of death/prediction of transplant-free survival and the relationship between NLR, NLR category (high/low), GAP Index, GAP Stage, age, sex, FVC (% predicted), TLco (% predicted), steroid therapy (as a binary variable), and transplant-free survival. Significance testing between groups on Kaplan–Meier curves was performed using non-parametric log-rank test. The log rank test was used to test the null hypothesis that there is no difference in survival between pre-specified groups (such as high vs low NLR). The ‘expected’ failure rates are what would be expected for each group if there was no difference in survival between the two, the ‘observed’ are the actual rates. Multivariate stepwise forward cox proportional hazards regression was used to determine whether NLR (as a continuous parameter or category) was independent of the GAP index/stage (and their individual components) and steroids in predicting patient transplant-free survival.

GAP Index-Plus and GAP Stage-Plus: For the NLR-modified GAP calculation, we proposed adding a fourth NLR variable that was binarized, as high (>/ = 2.9)/adverse (coded as 1) or favorable (<2.9) (coded as 0). This was then added to the existing GAP Index calculation where the modified GAP Index ranged from 0 to 9. For example, if a patient with original GAP Index “0” had a high NLR the modified GAP Index would be “0 + 1” = “1”. Conversely, if the patient with original GAP index “0” had a low NLR the modified GAP index would be “0 + 0” = “0”. So the “new” modified GAP index, which we called GAP Index-Plus ranged from 0 to 9 in comparison to the original GAP Index, which ranged from 0 to 8.

For GAP Stage-Plus we up-staged patients’ GAP stage by 1 if they were in the high NLR category. In this way we had a four category GAP stage such that original GAP Index of 0–3 = Stage 1; GAP Index of 0–3 plus high NLR = Stage 2; GAP Index of 4–5 = Stage 2; GAP Index of 4–5 plus high NLR = Stage 3; GAP Index of 6–8 = Stage 3; GAP Index of 6–8 plus high NLR = Stage 4.

The decision to have two different modifications that are not interchangeable was for ease of use for calculating GAP Stage-Plus or Index-Plus dependent on low (+0) or high (+1) NLR, as original GAP Index and Stage are both easily calculated by many available smartphone applications.

### Ethical approvals

Ethical approval was granted by the HRA and Health and Care Research Wales (HCRW) (REC reference: 18/LO/0937). Site specific and local R&D approvals were obtained at each participating site. Informed consent was not required for this anonymised, retrospective data.

### Role of the funding source

The funders had not input into the study design or interpretation.

Access to the data set is available by contacting the corresponding author.

The decision to submit the manuscript was made by JCP and TAM with agreement from all other authors.

## Results

Patient characteristics in the individual and pooled cohorts are summarised in Table 1 for demographic data available across the whole dataset. Data was not available across the whole data set for ethnicity, smoking status, BMI or other co-morbidities. For the 999 patients in the combined (discovery, validation and additional) cohorts, there were 533 events (death or transplant) recorded.

**Table 1:** Patient characteristics in derivation, validation and additional cohorts.

	Derivation cohort n = 71	Internal validation cohort n = 134	External Additional Exeter (all sites) n = 515	External Additional RBH n = 279	Combined all cohorts N = 999
Age years (SD)	71.05 (9.0)	74.7 (8.6)	74.0 (8.6)	69.6 (8.7)	72.7 (8.9)
Sex					
Male	62 (87.3%)	107 (79.9%)	380 (73.8%)	219 (78.5%)	768 (76.9%)
Female	9 (12.7%)	27 (20.2%)	135 (26.2%)	60 (21.5%)	231 (23.1%)
Lung function^a^					
FVC (%) (SD)	77.8 (17.9) n = 70	77.1 (21.1) n = 133	82.6 (20.2) n = 362	72.9 (16.6) n = 278	77.9 (19.5) N = 844
FEV1 (%) (SD)	78.0 (16.4) n = 69	82.5 (22.3) n = 123	86.0 (20.7) n = 329	77.2 (16.6) n = 266	81.8 (19.6) N = 788
bTLco (%) (SD)	44.8 (13.9) n = 61	47.9 (17.9) n = 114	49.9 (15.7) n = 274	41.9 (14.2) n = 258	46.2 (15.8) N = 707
TLco unable	8	12	1	21	42
TLco not done/recorded	2	8	240	0	250
GAP index mean (SD)	4.2 (1.6) n = 69	4.4 (1.5) n = 126	3.8 (1.3) n = 275	4.3 (1.4) n = 279	4.1 (1.4) N = 749
GAP stage					
1	20 (29.0%)	34 (27.0%)	124 (45.1%)	77 (27.6%)	255 (34.1%)
2	36 (52.2%)	65 (51.6%)	124 (45.1%)	143 (51.3%)	368 (49.1%)
3	13 (18.8%)	27 (21.4%)	27 (9.8%)	59 (21.2%)	126 (16.8%)
NLR					
Mean (SD)	3.9 (3.3)	4.1 (3.6)	3.5 (2.8)	4.6 (4.2)	3.9 (3.4)
Median (IQR)	2.9 (2.2–4.1)	3.1 (2.0–4.4)	2.8 (2.1–4.0)	3.2 (2.3–4.8)	2.9 (2.1–4.3)

a Lung Function was taken where available. In some cases, FVC was recorded without FEV1 (the latter is not part of GAP score).

b TLco: missing values were only included in GAPscore if documented as ‘not able to perform’- scoring 3 points. Not done or not recorded meant no GAP index was recorded.

The median NLR in the derivation cohort was 2.9 (95% CI, 2.2–4.1) and we used this cut-off to determine high (>/ = 2.9) or low NLR (<2.9). Median NLR across the additional cohorts was similar with UCLH validation cohort, 3.1 (2.0–4.4), Exeter additional cohorts 2.8 (2.1–4.0), and RBH additional cohort 3.2 (2.3–4.8). The combined cohort of 999 patients had a median NLR of 2.9 (2.1–2.3). When the original NLR cut-off of 2.9 was applied to the combined cohort, increasing age, male sex, and worse lung function parameters were all associated with the high NLR category (Table 2).

**Table 2:** Baseline and clinical characteristics of the patients (combined cohort, n = 999) in low (<2.9, n = 502) and high (>/ = 2.9, n = 497) NLR risk groups.

	Low NLR n = 502	High NLR n = 497	p value∗
Age years (SD)	71.9 (9.0) 95% CI (71.1–72.6)	73.5 (8.7) 95% CI (72.7–74.3)	**0.0054**
Sex			**0.009**
Female	134 (26.7%)	97 (19.5%)	
Male	368 (73.3%)	400 (80.5%)	
Spirometry			
FVC(%)	80.2 (SD 20.2) 95% CI (78.3–82.1)	75.5 (SD 18.5) 95% CI (73.7–77.3)	**0.0004**
FEV1 (%)	83.4 (19.7) 95% CI (81.5–85.4)	80.1 (19.3) 95% CI (78.2–82.0)	**0.016**
TLco % predicted (SD) (n = 707)	48.8 (15.4) 95% CI (47.2–50.4)	43.5 (15.7) 95% CI (41.9–45.2)	**<0.0001**
GAP index	3.9 (1.4) 95% CI (3.7–4.0)	4.4 (1.4) 95% CI (4.2–4.5)	**<0.0001**
GAP stage	N = 372	N = 377	**<0.0001**
1	154 (41.4%)	101 (26.8%)	
2	170 (45.7%)	198 (52.5%)	
3	48 (12.9%)	78 (20.7%)	

∗p values were calculated by unpaired two-tailed t-test except for sex and GAP stage where a Fisher's exact test was used.

For the derivation cohort (n = 71) there was a significant difference in the median survival between high NLR (>/ = 2.9) or low NLR (<2.9) with median survival of 62.1 months (IQR 20.2-na), in the low NLR group (n = 36), versus 24.3 months (IQR 11.4–69.8) in high NLR (n = 35), p = 0.0125. This increased mortality was confirmed in the validation cohort (n = 134) with median survival in the low NLR group (n = 64) of 46.5 months (IQR 16.8–93.50) and in the high NLR group (n = 70) of 16.9 months (IQR 9.7–43.4), p = 0.0125 (Table 3).

**Table 3:** Median survival in low (<2.9, n = 502) and high (>/ = 2.9, n = 497) NLR risk groups per study site/cohort ∗ p values were calculated by two-sided Fisher's exact test.

	Derivation cohort UCLH n = 71	Internal validation cohort UCLH n = 134	External additional SW&L n = 515	External additional RBH n = 279	UCLH validation plus external N = 928	Combined Cohort N = 999
**Low NLR**	n = 36	n = 64	n = 297	n = 120	n = 466	N = 502,
Median survival months (IQR)	62.1 (20.2-na)	45.3 (16.8–93.5)	57 (32–157)	46.5 (22.6–80.0)	49.6 (25.9–89.5)	49.8 (24.8–88.3)
Incident rate	0.013	0.017	0.011	0.017	0.013	0.013
Total time at risk (months)	1337.0	1630.7	9344	5784.0	16370.7	17,707
**High NLR**	n = 35,	n = 70	n = 218	n = 159	n = 462	N = 497
Median survival	24.3 (11.4–69.8)	16.9 (I9.7–43.4)	44 (18–121)	39.8 (19.8–58.8)	39.0 (16.0–63.7)	35.9 (15.1–63.7)
Incident rate	0.026	0.031	0.016	0.023	0.021	0.021
Total time at risk (months)	975.6	1226.5	5479	6356.6	13450.8	14,426
P value	0.0255	0.0125	0.0037	0.0223	<0.0001	<0.0001

For each of the individual external cohorts the improved survival with low NLR was consistent (Table 3). SW&L: median survival low NLR (n = 297) of 57 months (IQR 32–157), high NLR (n = 218) of 44 months (IQR 18–121) p = 0.0037; RBH cohort, median survival low NLR (n = 120) of 46.5 months (IQR 22.6–80), high NLR (n = 159) of 39.8 months (IQR 19.8–58.8) p = 0.0223 (Table 3). The same was seen for the combined validation and external cohorts (n = 928 (excluding discovery), median survival low NLR (n = 466) of 49.6 months (IQR 25.9–89.5), high NLR (n = 462) of 39 months (IQR 16–63.7) p=<0.0001.

**Fig. 1:**
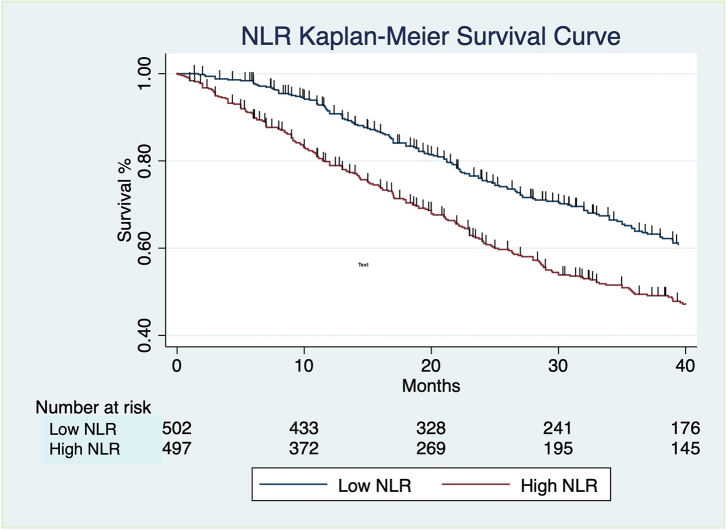
**Kaplan–Meier survival curve for all-cause mortality following a diagnosis of IPF for patients in low (<2.9, n = 502) and high (>/ = 2.9, n = 497) NLR category at baseline, with follow up of 40 months**. The numbers of patients at risk at 10, 20, 30, 40 months for each of these groups is shown in the table immediately below the survival curves This demonstrates a significant difference in mortality between high and low categories (log rank test, p < 0.0001).

Finally, the patients were taken as a combined cohort of 999 and were divided into high NLR (>/ = 2.9) or low NLR (<2.9) at time 0; there was a significant difference in the median survival between high and low NLR groups (Fig. 1; p < 0.0001). Median survival in the low NLR group (n = 502) of 49.8 months (IQR 24.8–88.3), incident rate of 0.013 and a total time at risk of 17,707 months; median survival in the high NLR group (n = 497) of 35.9 months (IQR 15.1–63.7, incident rate of 0.021 and a total time at risk of 14,426 months (Table 3 and Fig. 1).

We then used this combined cohort of 999 patients, to investigate whether the addition of NLR could refine the GAP clinical scoring. We first showed that the association of NLR category with GAP stage or GAP Index was highly significant (Table 2; p < 0.0001). Although gas transfer data was only available for 71% of subjects, a lower TLco was significantly associated with high NLR (42.2% pred. versus 47.7%, p < 0.0001).

For this combined patient cohort, the observed mortality was similar to that predicted using GAP stage predicted mortality^16^; (Supplementary Table S1. Median survival per GAP stage is summarised in Table 4. Median survival as stratified by NLR risk category was not significantly different for GAP stage 1 (p = 0.245) or 3 (p = 0.1381) but was significant for GAP stage 2 (p = 0.0127) and for the remaining patients who had no GAP stage recorded due to insufficient lung function data (p = 0.0015; Table 5).

**Table 4:** Median survival per GAP stage for combined cohort (n = 999 of which GAP not recorded in n = 250).

GAP Stage n = 749	Incidence rate	Median survival in months (IQR)
GAP 1 n = 255	0.010	73.7 (36.0–100.9)
GAP 2 n = 368	0.020	40.7 (19.5–60.0)
GAP 3 n = 126	0.037	18.0 (10.0–33)
GAP unrecorded n = 250	0.013	60 (22–120.6)

**Table 5:** Median survival per GAP stage stratified for low (<2.9) and high (>/ = 2.9) NLR category, n = 999 of which n = 250 had unrecorded GAP.

GAP Stage and NLR category N = 999	Incidence rate	Median survival months	Recorded failures	p- value∗
GAP 1 NLR low n = 154	0.009	76.1 (41.8–100.9)	61	
GAP 1 NLR high n = 101	0.011	55.8 (33.2–92.5)	48	.245
GAP 2 NLR low n = 170	0.017	44 (22.3–65.0)	94	
GAP 2 NLR high n = 198	0.023	35.7 (16.6–54.2)	137	**0.0127**
GAP 3 NLR low n = 48	0.031	19.5 (13.0–39.4)	41	
GAP 3 NLR high n = 78	0.042	16.9 (8.2–33)	67	.1381
GAP unrecorded NLR low n = 130	0.009	83 (34–120.6)	39	
GAP unrecorded NLR high n = 120	0.018	44 (12–74)	51	**0.0015**

∗2-sided Fisher's exact.

**Fig. 2:**
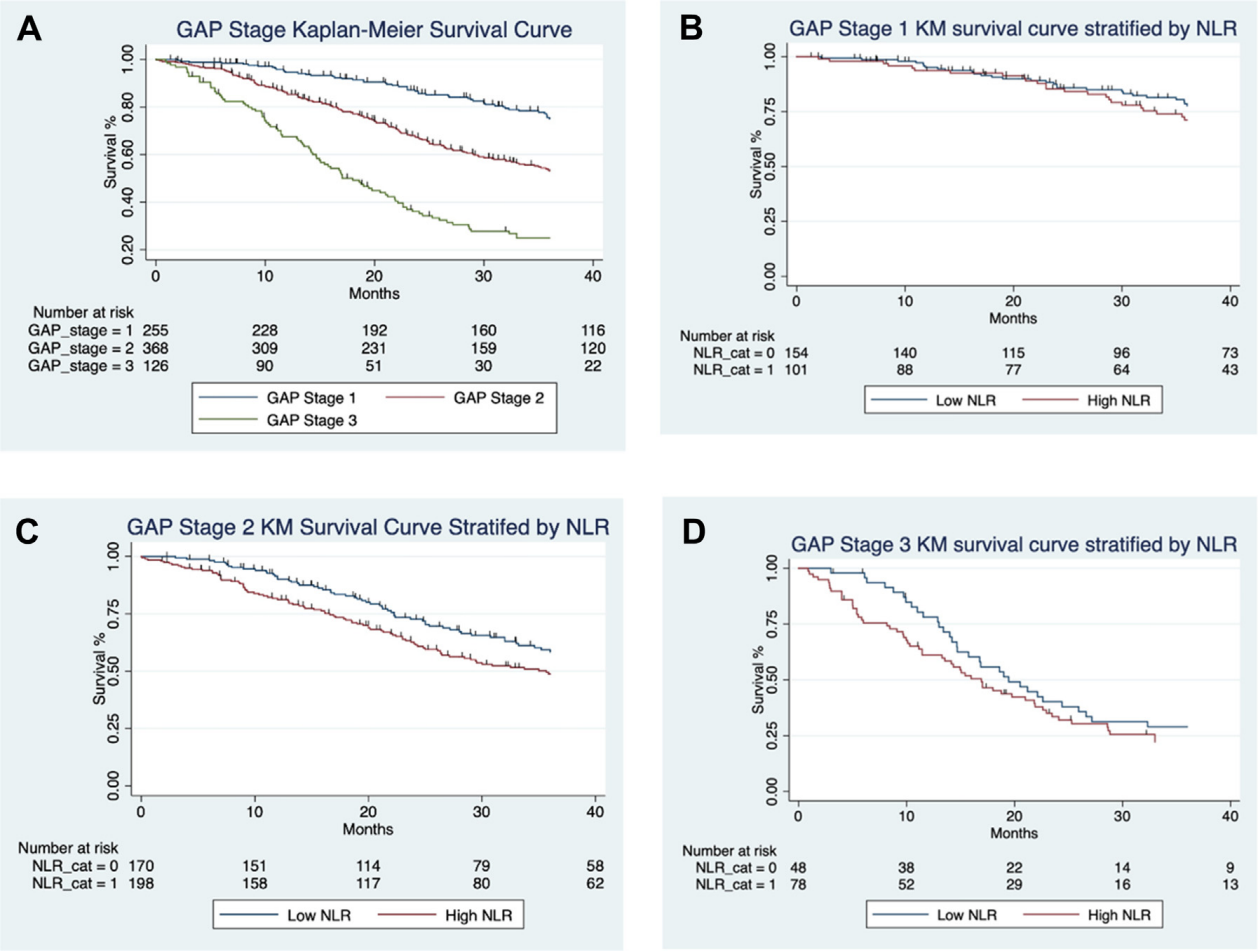
**Kaplan–Meier survival curves for all-cause mortality following a diagnosis of IPF with follow up extending to 40 months**: A, All patients in combined cohort (n = 999) divided into GAP stages 1 (n = 255), 2 (n = 368) and 3 (n = 136); B, Patients in GAP Stage 1 stratified into low (<2.9, n = 154) and high (>/ = 2.9, n = 101) NLR category at baseline; C, Patients in GAP Stage 2 stratified into low (<2.9, n = 170) and high (>/ = 2.9, n = 198) NLR category at baseline; D, Patients in GAP Stage 3 stratified into low (<2.9, n = 48) and high (>/ = 2.9, n = 78) NLR category at baseline. The numbers of patients at risk at 10,20,30,40 months for each of these groups is shown in the table immediately below the survival curves. Differences between survival in patients with different GAP stages 1–3 (A) reached significance (log rank test, p < 0.0001). Stratifying patients in the same GAP stage by NLR category (low/high) only showed significant differences in survival between low and high NLR for GAP stage 2 (log rank test, p < 0.0001; (C)), and not for GAP stage 1 (log rank test, p = 0.1755; (B)), or stage 3 (log rank test, p = 0.0871; (D)).

The difference in expected versus observed events, based on log-rank test for equality of survivor functions, for patients in high and low NLR categories in the combined cohort (n = 999) was significant with 235 observed events out of 300.95 expected in the low NLR group, versus 303 observed events out of 237.05 expected in the high NLR group (log rank test, p < 0.0001; Fig. 1). Differences between survival in patients with different GAP stages 1–3 (Fig. 2A) and GAP Index scores (not shown) reached significance (log rank test, p < 0.0001). Stratifying patients in the same GAP stage by NLR category (low/high) only showed significant differences in survival between low and high NLR for GAP stage 2 (log rank test, p < 0.0001; Fig. 2C), and not for GAP stage 1 (log rank test, p = 0.1755; Fig. 2B), or stage 3 (log rank test, p = 0.0871; Fig. 2D).

**Fig. 3:**
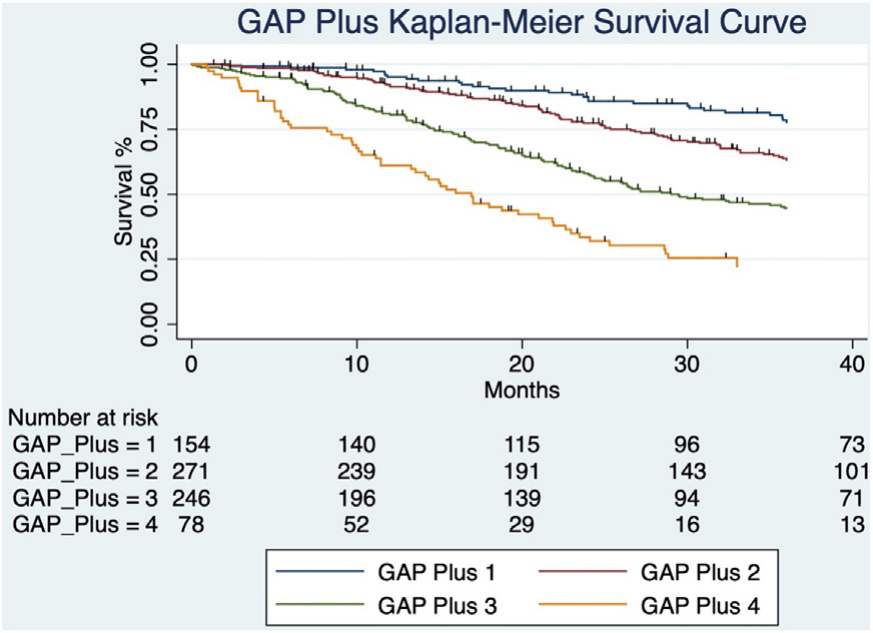
**Kaplan–Meier survival curves for GAP Stage-plus categories show all-cause mortality following a diagnosis of IPF with follow up extending to 40 months**: A, All patients in combined cohort (n = 999) were assigned to GAP Stages-plus (initial GAP Stage plus an additional 1 for patients with high, >/ = 2.9, NLR category at baseline): GAP-plus stage 1 (n = 154), 2 (n = 271) 3 (n = 246) and 4 (n = 78); The numbers of patients at risk at 10, 20, 30, 40 months for each of these groups is shown in the table immediately below the survival curves. The survival differences between the GAP Stage-plus groups of patients reached significance (HR 1.80, 95% CI 1.60–1.98; log rank test, p < 0.0001).

We proposed an NLR-modified GAP calculation, GAP Index-plus and GAP Stage-Plus (see methods) using a very simple modification of GAP dependent on low (+0) or high (+1) NLR, which was memorable and easily used. Survival differences between groups were significant for both GAP Index-plus (HR 1.4, 95% CI 1.29–1.51, p < 0.0001; figure not shown) with survival differences between the GAP Stage-plus groups of patients also reaching significance (HR 1.80, 95% CI 1.60–1.98; log rank test, p < 0.0001; Fig. 3).

**Fig. 4:**
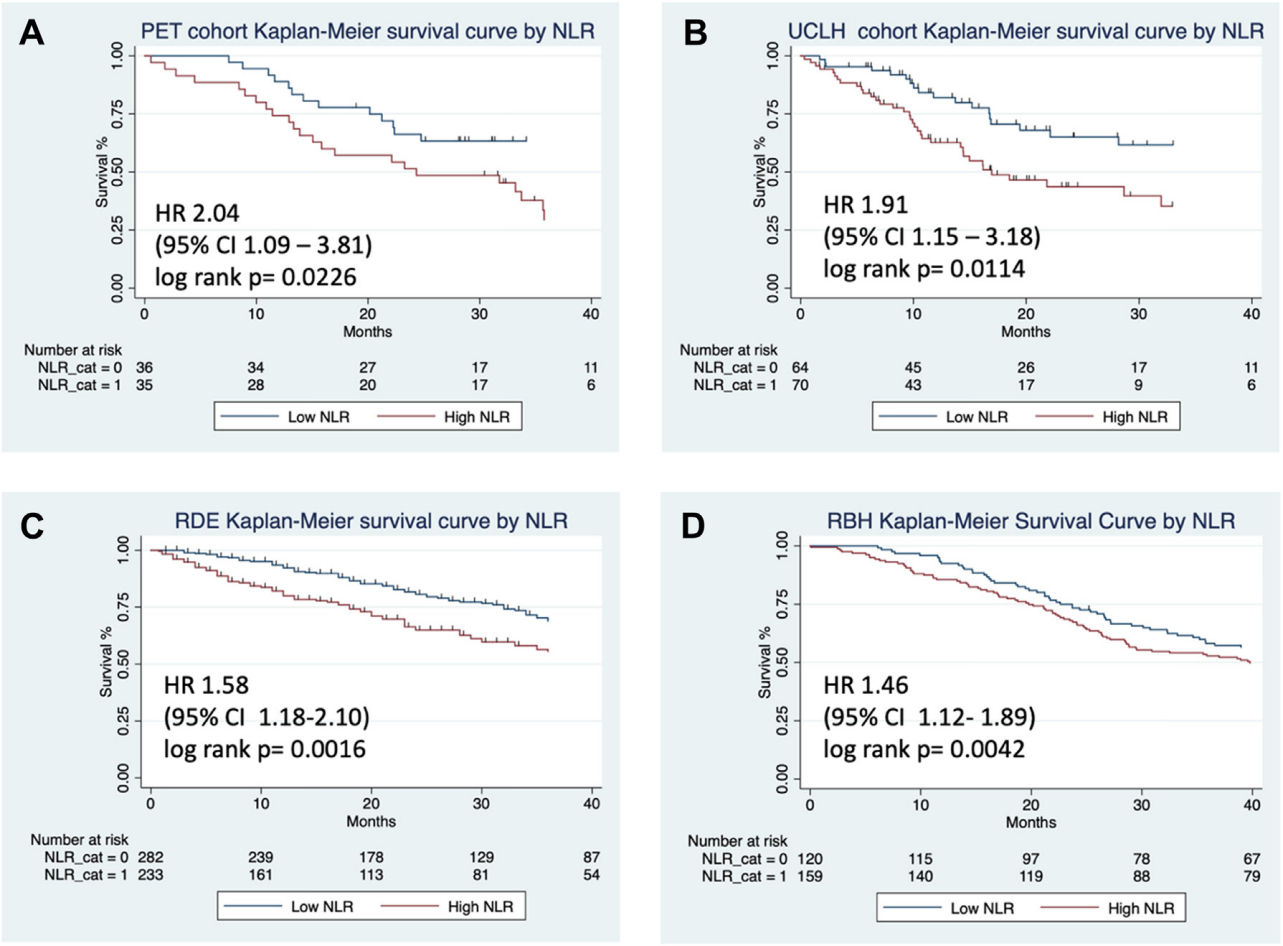
**Kaplan Meir survival curves for NLR categories for cohorts shown for each centre show all-cause mortality following a diagnosis of IPF with follow up extending to 40 months. Graphs show patients in each cohort**: A, derivation cohort, UCLH (n = 71); B, validation cohort at UCLH (n = 134); C, validation cohort RDE (n = 515); D, Validation cohort RBH (n = 279) who were assigned to low (<2.9) or high (>/ = 2.9) NLR category at baseline: The numbers of patients at risk at 10, 20, 30, 40 months for each of these groups is shown in the table immediately below the survival curves. HR, hazard ratio for event is shown for patients with high NLR compared to low NLR and include CI, confidence intervals, with P values for log rank tests shown on individual plots.

Univariate Cox proportional hazards models of the combined cohort (n = 999) showed that patients in the high NLR category group had significantly higher mortality/progression to lung transplant when compared with patients in the low NLR group (HR 1.65, 95% CI 1.39–1.95; p < 0·0001; not shown), reflecting their baseline demographics (Table 2). NLR category remained significant when each site's cohort was considered individually (Fig. 4). Analysis was repeated in the combined cohort excluding all patients on known steroid therapy with a comparable result (HR 1.50, 95% CI 1.24–1.82; p < 0.0001; data not shown). Univariate regressions for GAP Index, GAP Stage, GAP Index-plus and GAP Stage-plus were all significant (GAP index, HR 1.4, 95% CI 1.3–1.5, p < 0.0001; GAP Stage, HR 2.1, 95% CI 1.8–2.4, p < 0.0001; GAP Index-plus, HR 1.4, 95% CI 1.29–1.51, p < 0.0001; GAP Stage-plus HR 1.8, 95% CI 1.6–2.0, p < 0.0001). Univariate regression was carried out for all the individual GAP components (age, sex, FVC % pred. TLco % pred.) and was significant for all except sex. Age, HR 1.02, 95% CI 1.1–1.03, p < 0.0001; sex, HR 1.2, 95% CI 0 .99–1.51, p = 0.065; FVC% pred, HR 0.98, 95% CI 0.97–0.99, p < 0.0001; TLCO% pred, HR 0.97, 95% CI 0.96–0.97, p < 0.0001. There was significant difference in the individual components of GAP (age, FVC% pred, TLco% pred but not gender) between patients with high and low NLR based on non-parametric Wilcoxon rank-sum test (Age, p = 0.003; FVC% pred, p = 0.0023; and TLco% pred p < 0.0001). Cox regression for steroid use was also significant for transplant-free survival (HR 1.71, 95% CI 1.37–2.12 p < 0.0001). The analysis was then repeated in this cohort but with the exclusion of all those patients who had ever taken oral steroids and showed the same significances.

Multivariate analysis was then performed using these individual components as covariates within the model: age, sex, FVC%, TLco%, GAP Stage, use of steroids, NLR (continuous or binary high/low). This analysis showed that after adjusting for GAP Stage and use of steroids in the combined dataset a high NLR category was independently predictive of mortality/progression to lung transplant (HR 1.36, 95% CI 1.12–1.66; p = 0.002). Repeating the analysis using the individual GAP components (age, sex, FVC%, TL_CO_%) as variables and again adjusting for steroids showed similar results (HR 1.26, 95% CI 1.03–1.55; p = 0·027). Inputting NLR as a continuous variable was also independently predictive adjusted for the individual GAP components and steroids (HR 1.04, 95% CI 1.01–1.07; p = 0.011) as well as when adjusted for GAP stage and steroid use (HR 1.05, 95% CI 1.02–1.07, p = 0.001). All GAP components, except for sex, continued to be significant when adjusted for each other, NLR and steroid use.

Although, for most of the patients, the baseline CBC predated the use of antifibrotics, some patients were later started on antifibrotics. Patients who had taken antifibrotics for >6 weeks were identified in the Southwest (RD&E, MPH, NBT; n = 415) and RBH (n = 270) cohorts. In Southwest cohort 275 of 415 (66.3%), and in RBH cohort 231 of 270 (85.6%) patients were recorded to have taken antifibrotics. Therefore, of a sub-cohort of 685 patients, 606 (73.9%) had taken antifibrotics. Univariate regression for antifibrotic therapy was not significant for mortality (HR 1.01, 95% CI 0.79–1.29, p = 0.95). Multivariate regression taking into consideration NLR category and antifibrotic therapy showed that antifibrotic use remained a non-significant predictor (HR 1.02, therapy 95% CI 0.79–1.30, p = 0.899), whereas NLR category was significant (HR 1.59, 95% CI 1.30–1.95, p < 0.0001).

Harrell's concordance (C)-index prediction accuracy confirmed that the best performing prediction model was based on the component variables making up the GAP, with NLR as a continuous variable and adjusted for steroids. GAP index was clearly better than GAP stage, but incorporating NLR into GAP staging as GAP Index-Plus further increased the model's ability to predict patient mortality (C-index 0.673, 0.645–0.701; Table 6).

**Table 6:** Harrell's C-index performance in the pooled patient cohort (n = 999) including steroid users in various predictive models.

Cox regression model	C-Index	95% CI
UNIVARIATE		
GAP Stage	0.652	0.627–0.678
GAP Index	0.666	0.639–0.694
NLR Category	0.574	0.550–0.597
NLR	0.610	0.584–0.637
GAP Stage Plus	0.659	0.633–0.686
GAP Index Plus	0.673	0.645–0.701
MULTIVARIATE		
GAP Stage NLR category	0.670	0.643–0.697
GAP Stage NLR	0.680	0.652–0.708
NLR, age, sex, FVC% predict, TLCO% predict, steroids	0.711	0.683–0.740
NLR, age, sex, FVC% predict, TLCO% predict	0.708	0.679–0.736
NLR category, age, sex, FVC% predict, TLCO% predict, steroids	0.708	0.680–0.737
NLR category, age, sex, FVC% predict, TLCO% predict	0.705	0.677–0.734
NLR category, age, sex	0.61	0.59–0.64
Age, sex	0.59	0.56–0.61

**Fig. 5:**
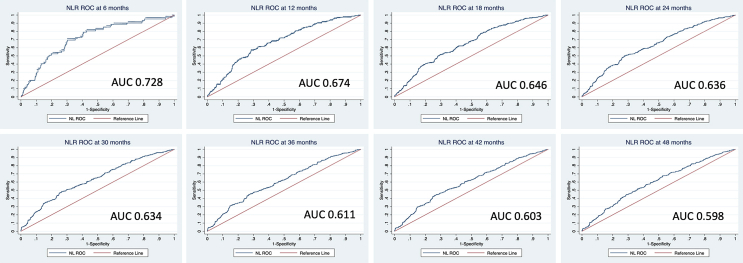
**Ability of baseline NLR to predict all-cause mortality in patients with IPF decreases with time**. Time dependent change of AUC and ROC in NLR at 6, 12, 18, 24, 30, 36, and 42 months: AUC, area under the curve; ROC, receiver operator characteristic.

Time-dependent ROC analysis in the pooled cohort for NLR demonstrated the continuous decline of the model's predictive value with the passing of time. For example, AUC at 6 months is 0.728 which declines to an AUC of 0.598 at 48 months (Fig. 5).

## Discussion

IPF is a devastating disease with a variable clinical course. One of the most used prognostic cohort scoring systems is the GAP score. However, even within the same GAP stage, and particularly for moderately severe GAP stage II, patients may have very heterogenous outcomes. This has led to a concerted effort to identify better tools for individual patient risk stratification. The ideal biomarker would be measurable in the serum using a simple and widely available test and would predict prognosis and potentially response to therapy. Here we show that baseline NLR derived from a cheap and widely available routine blood test, identifies two groups of patients with IPF with significant differences in outcome. We go on to show that NLR can significantly refine the predictive capacity of the clinical GAP index.

The search for viable biomarkers has taken advantage of the rapidly expanding knowledge of IPF immunopathogenesis. Aberrant repair processes initiated by repetitive injury to the alveolar epithelium result in an exaggerated tissue remodelling response and fibrosis of the lung parenchyma. Proteins released from damaged epithelium and collagen degradation products can enter the systemic circulation, acting as markers of disease activity by proxy-the most promising of which include CA-19-9,^18^ CA-125^18^ and CCL18.^19^ Others that have been investigated include SP-D,^20^ MMP7,^21^ osteopontin (OPN), periostin (PON), ICAM1^22^ and telomere length.^23^ In addition, neo-epitopes generated by the action of matrix metalloproteinases (MMPs) on collagen can be detected in the serum and Jenkins et al. found that 6 of 12 of these were predictive for mortality.^24^ Other serum biomarkers include CD28, ICOS, LCK, ITK^25^ alone or as part of a 52-gene RNA signature.^1^ More recently, attention has turned to imaging biomarkers including imaging quantification,^26^ measurements of glucose uptake in the lung with Positron Emission Tomography (PET)^2^ and a combination of the two.^27^

However, of these, only three biomarkers have been validated to refine the GAP staging system by identifying high and low risk patients within a given GAP stage. The 52-gene expression signature, an approach that requires calibration against a control cohort,^1^ a composite of OPN, PON, MMP-7 and ICAM-1^22^ and the Total-to-Background Ratio (TBR) calculated from 18F-FDG-PET imaging, with only the 52-gene expression validated in multiple independent cohorts.

NLR which is calculated from complete blood count with differential, is an inexpensive, easy to obtain, widely available and emerging marker of disease activity and prognosis in patients with chronic inflammatory diseases, cardiovascular diseases, and malignancies.

Previous studies have specifically considered NLR as a biomarker in IPF: Of two small single centre studies; the first^28^ found NLR raised in 21 patients with IPF compared to 42 healthy controls but was not prognostic; the second study of 73 patients with IPF and 62 healthy controls found that NLR and monocyte lymphocyte ratio (MLR), but not platelet to lymphocyte ratio (PLR), associated with IPF and correlated negatively with FVC/TLco.^29^ Another study measured NLR in bronchoalveolar lavage (BAL) samples from 59 patients with IPF and found that BAL NLR was inversely correlated with FVC measured at the same time as collection of the BAL sample.^30^ We presented our discovery and validation cohort of 218 patients in 2018.^14^ Our initial findings were taken further by Nathan et al.,^12^ who included 1334 patients involved in ASCEND (Study 016; NCT01366209) and CAPACITY (Studies 004 and 006; NCT00287716 and NCT00287729) as a discovery cohort and placebo-treated IPF patients from two independent Phase III, trials of IFN-γ-1b (GIPF-001 (NCT00047645) and GIPF-007 (NCT00075998) as a validation cohort. Significant trends were observed between baseline NLR and PLR quartiles for various outcomes including: physiological decline; respiratory hospitalization; and all-cause mortality. However, the only consistent correlation in the discovery cohort was with baseline NLR and the composite endpoint of ‘absolute decline in 6MWD ≥50 m or death’ at 12 months, a finding that was not tested against the validation cohort. Alongside this other groups were investigating circulating cellular biomarkers in IPF. Significant prognostic effects were found for monocyte count a finding validated in >7000 patients with IPF from five independent cohorts^31^ and >2000 patients from a further four cohorts.^32^ However, the ability of monocyte count to enhance the predictive accuracy of GAP, although promising has not been validated^33^ in clinical cohorts.

In this retrospective study, we have extended the findings of Nathan et al. to investigate the role of NLR in multiple ‘real-life’ IPF cohorts with a longer follow-up period, to see if the current clinical prediction GAP score could be further refined. We analysed the NLR in a derivation cohort of patients and identified a median value of NLR that separated our discovery population into a high and low risk group for transplant-free survival with significant differences in mortality. We then investigated the prognostic ability of this NLR cut-off in an internal validation cohort of IPF patients and then in a combined cohort which included the addition of two further IPF cohorts provided by five other ILD specialist service centers in the UK. Furthermore, we showed, using a variety of statistical models, that the NLR is an independent risk factor for mortality, and addition of NLR risk profiles to further refine GAP index cohorts significantly increased the prediction accuracy of this clinical score. Although NLR is, unsurprisingly, even more highly predictive as a continuous measure than as a binary ‘high’ or ‘low’, our aim was to modify the GAP score in a simple memorable way, and so we opted for a simple modification of GAP (+1 for ‘high’; +0 for ‘low’) rather than to create a complex composite score in which absolute NLR is incorporated into GAP.

We went on to show that the addition of NLR data to GAP score refines the existing mortality prediction model by using C-index and ROC statistics. As expected, the more granular the data inputted the better the prediction model, hence the increased C-Index for a model using the individual components of the GAP Index rather than an overall score. This despite marked heterogeneity between the cohorts, with the SW&L cohort being more recent (2011–2019) with a lower average GAP Index, GAP Stage and mean, and median NLR compared with the other cohorts. It is encouraging that NLR mortality prediction was robust despite this heterogeneity, pointing to wide-spread applicability.

However, we should emphasise that although the use of GAP scoring with the addition of the binary high/low NLR provides an easily applied tool to establish clinical cohorts of patients; GAP/NLR, although an improvement on GAP alone, is still limited when used for individual, rather than cohort, prognostication. The C-index, although improved is still only 0.71 which is lower than other biomarkers used for clinical decision making. A more robust approach for an individual patient would ultimately necessitate input of more granular data, an approach that underlies scoring systems such as the composite physiological index (CPI).^34^

By using time-dependent ROC analysis we were able to calculate the decline in NLR's predictive accuracy over time and establish that it is most accurate shortly after being measured, a time when indeed it is most useful. Many newly diagnosed patients are keen to discuss prognosis early and as clinicians we often refer to variable disease trajectory and the need to observe lung function over time to allow more accurate prognostication. However, these data suggest that even early mortality might be predictable from a high NLR at presentation and may expedite, for example, lung transplant assessment in appropriate patients. A similar decline in predictive accuracy with time has been shown for GAP and other biomarkers^1^^,^^16^

The difference in median survival stratified by GAP stage was only significant for patients in the moderately severe GAP stage 2 (n = 368) and in those patients in whom the GAP stage could not be calculated (n = 250). This probably reflects the small number of events for patients at GAP stage 1 and the small number of patients at GAP stage 3 (n = 126), although similar trends to significance in these groups are encouraging. In those patients in whom GAP could not be calculated it was interesting to observe that the overall median survival of 60 months was between the median survivals for GAP Stages 1 (73.7 months) and GAP Stage 2 (41 months). When the patients in GAP Stage 2 were stratified according to NLR category, a remarkable difference of survival became apparent on either side of this median with Gap Stage 2 and low NLR having a median survival of 83 months, and almost double that of those in GAP Stage 2 with a high NLR whose median survival was just 44 months. Although less helpful for mild (stage 1) or very ill (stage 3) patients, the ability of a combined NLR/GAP score to further refine those moderately severe IPF patients (stage 2), is particularly helpful as: stage 2 is the more frequently represented stage; outcomes of stage 2 are the most heterogenous and it is in this patient group that clinical decision making can be most difficult.

GAP staging was not possible for those patients with incomplete lung function data, nearly always due to missing TLco readings. Gas transfer may be missing for several reasons, either the patient is unable to perform the test hence coded as a “3” (maximum) in the GAP index or a data quality issue. We found that patients with no TLco but in the low NLR group had a longer median survival than other patients in GAP stage 1, indicating that this subgroup may have been too well to warrant full lung function work up at the time of presentation. One additional feature of this study is our demonstration that NLR correlates with lung function, suggesting NLR may offer a cheap and quick screening test to fast-track high risk patients for early tertiary care review and urgent lung function. In fact, NLR as a continuous variable was almost as predictive as GAP score (Table 6: C-index of 0.66 for GAP Index versus 0.6 1 for NLR) and potentially easier to generate as there is no reliance on lung function. Table 6 also shows that NLR (high/low) can refine scoring based on age and sex alone. If faced with limited lung function testing ability it might be prudent to prioritise IPF patients at highest risk based on age, sex and NLR. This might be especially relevant in current times of increased pressure and backlog on lung function testing due to the COVID-19 pandemic and in remote, and resource poor, areas where access to lung function is restricted. In addition, lung function can be influenced by operator, equipment, and patient factors such as sub-optimal maneuvers whereas CBC analysis maintains objectivity.

It is unclear why NLR is raised in patients with IPF with decreased survival. We propose it could be a marker for ongoing inflammation. The term interstitial lung disease or ILD covers a group of over 200 different diseases with varying degrees of inflammation and/or fibrosis. It is unclear whether fibrosis is always preceded by inflammation, although this is more likely to be true for ILDs associated with underlying autoimmune rheumatic diseases. In such situations, NLR has been shown to predict development and extent of lung fibrosis, for example in systemic sclerosis,^10^ and dermatomyositis/polymyositis.^11^ In this study, we demonstrate that NLR is also predictive in IPF, a disease in which inflammation is not thought to play a role, and indeed in which the use of immunosuppression in this disease has been shown to be harmful. It is unclear if NLR is alerting us to a potential role of inflammation in advancing interstitial inflammation or is highlighting a group of patients in which inflammation drives increased mortality from cardiovascular involvement. Disordered metabolism of carbohydrates, lipids, proteins, and hormones has been documented in lung, liver, and kidney fibrosis and metabolic dysregulation has been implicated in the pathogenesis of IPF,^35^ potentially offering a new target for fibrosis therapy.

The predictive ability of NLR may hint more directly at a role for neutrophilic inflammation in the pathogenesis of IPF. We have known for a long time that the percentage of neutrophils in the BAL of patients with IPF correlates with a poor outcome.^36^ Molyneaux et al. have shown that BAL neutrophilia is associated with both increased microbiome burden and progressive IPF,^37^ with subtle changes in the microbiome implicated in the initiation and progression of IPF in the absence of identified infection.^38^ The increased bacterial burden of IPF appears to be in the airway, proximal to the actual fibrotic remodelling of the parenchyma, with very low levels of bacteria identified in IPF parenchymal lung tissue.^39^ However, such changes are unlikely to cause increases in systemic neutrophilia and NLR in the absence of overt infection. In our study, we excluded patients diagnosed clinically with infection and started on antibiotics, and those in whom the C reactive protein (CRP) was greater than 20 mg/L.

If we do not think NLR is detecting occult infection, then why is it such a powerful marker? One developing line of enquiry is that the lung is responsible not just for gas-exchange but also plays a crucial role in leukocyte homeostasis. There is increasing evidence that the lung may orchestrate the disposal of aged neutrophils, by targeting them for recirculation to, and disposal in, the bone marrow. In a mouse model the inability of the lung to clear aged neutrophils resulted in a pulmonary fibrosis.^40^ As well as neutrophil activation, other groups have noted phenotypic changes in circulating leukocytes, for example CD28 downregulation on CD4 cells, perhaps reflecting T cell exhaustion, and 4 T cell genes (CD24, ICOS, LCK and ITK) are part of the 52 gene signature that is associated with a poor disease outcome.^1^ Interestingly we found that the neutrophil count is not as strong a predictor of mortality in IPF as NLR, suggesting that both neutrophil activation and lymphocyte exhaustion may be relevant.

Despite the reproducibility of our findings there are some caveats. We did not determine the specificity of NLR to IPF as opposed to other ILDs. However, we have previously reported that within the ILD cohort from RDE/NB/TS/UHL although high baseline NLR predict outcomes in IPF this was not the case in patients with chronic hypersensitivity pneumonitis.^15^ Secondly, most of the patients were in the pre-antifibrotic era and many were treated with corticosteroids although we lack granular information on the doses and duration of such treatments. However, we have shown that neither the use of corticosteroids nor of antifibrotics influenced either patient outcomes or the validity of NLR. Although surprising, this likely reflects: the small number of patients in these subgroups combined with the heterogenicity of our study populations when compared to clinical trial cohorts, making it underpowered to pick up the predicted favourable outcome with antifibrotics^41^ or worse outcome with steroids.^42^^,^^43^ In addition, it is possible, although unproven, that these cohorts were exposed to lower doses (<20 mg) of prednisolone, compared to the doses of 0.5 mg/kg, average of 30 mg prednisolone, that were shown to be harmful in the PANTHER^42^ and other^43^ studies in which no excess adverse signal was seen once dose was reduced to 20 mg.^44^ The fact that despite this heterogeneity the prognostic potential of NLR still holds is encouraging. We have only limited longitudinal data, and there is a suggestion that patients will change their profiles but how this relates to their prognosis remains unclear. Nathan et al. found NLR change may be an even more robust prognostic biomarker than baseline NLR but may be less suitable as a predictive biomarker for patients receiving treatment with antifibrotics.^12^ The main limitation of this retrospective study is linked to missing, and at times, poor quality data. In particular, we were lacking basic demographic data such as ethnicity, smoking status, and co-morbidities that were not consistently available across all cohorts. In addition, although all cases were incident IPF and CBC was measured at first appointment of IPF diagnosis, we did not consider time to diagnosis which has been shown to vary considerably in UK.^45^ However, our data does offer impetus to the idea that NLR should be evaluated as part of a prospective clinical trial as a secondary or an exploratory endpoint.

In summary, we have demonstrated and validated that NLR, an easily, widely available, cheap and reproducible test, is an independent prognostic biomarker that can be evaluated at diagnosis in patients with IPF and may inform future management of these patients. There is an enhanced cohort outcome prediction accuracy when NLR is added to GAP score suggesting that NLR may be useful not only as a stratification marker, but also a predictor for disease monitoring in IPF. One striking observation is that NLR correlates with lung function (FVC and TLco) and may be particularly helpful in assessing clinical priorities in situations where lung function is not easily available, such as in remote areas and during the pandemic, or cannot be performed by the patient. Further evaluation of the utility of NLR measurement for therapeutic decision making is warranted.

## Contributors

Literature search and conceptualisation JCP and TAM. Figures TAM.

Study design TAM, JCP, CJS. Data collection and curation: all authors. Data analysis: TAM, PG, JS, BG, CS, JP Data interpretation: all authors. Writing – first draft: TAM and JCP. Review, editing and final approval: all authors.

## Data sharing statement

Data collected for the study may be accessed after approval of a proposal and with a signed data access agreement with the individual investigators that manage the patient databases.

## Declaration of interests

SLB reports consultancy fees from Boehringer Ingelheim (BI). PMG reports personal fees from BI and AstraZeneca (AZ) and Brainomix and lecturing honoraria from BI, Roche and Cipla. VK reports lecturing fees from Novartis, Roche, and BI. JCP reports consulting fees from Carrick therapeutics, AZ and lecturing honaria from The Limbic. EAR reports lecturing fees from BI and Mundipharma. SL reports conference attendance support from BI. AUW reports honoraria from BI and Roche and consulting fees from Roche, BI and Veracyte. FAW reports support for conference attendance from BI. FW is now a full-time employee of Avalyn Pharma Inc, but all work related to this manuscript was carried out whilst a fulltime NHS employee of the University Hospitals of Leicester NHS Trust. All other authors have nothing to disclose.
