# Supplementary material for: Multi-center evaluation of baseline neutrophil-to-lymphocyte (NLR) ratio as an independent predictor of mortality and clinical risk stratifier in idiopathic pulmonary fibrosis
Source: eClinicalMedicine. 2022 Dec 1;55:101758. doi: 10.1016/j.eclinm.2022.101758 (PMC9722446; doi:10.1016/j.eclinm.2022.101758)
Supplement: Supplementary File S1 [file mmc1.docx]

**Supplementary Table**

**Table S1: Observed (and Predicted) Mortality by GAP Stage for our cohort (n=999) compared to the literature predicted values**

| Observed Mortality by GAP Stage compared with Predicted  in Pooled Study Cohort | | | |
| --- | --- | --- | --- |
| Stage | **1-year** | **2-year** | **3-year** |
| I  (n=255) | 5.3%  (predicted 5.6%) | 13.2%  (predicted 10.9%) | 24.2%  (predicted  16.3%) |
| II  (n=368) | 13.7%  (predicted 16.2%) | 32.5%  (predicted 29.9%) | 46.3%  (predicted 42.1%) |
| III  (n=126) | 32.4%  (predicted 39.2%) | 64.1%  (predicted 62.1%) | 75.1%  (predicted  76.8%) |
