# Supplementary material for: Multi-center evaluation of baseline neutrophil-to-lymphocyte (NLR) ratio as an independent predictor of mortality and clinical risk stratifier in idiopathic pulmonary fibrosis
Source: eClinicalMedicine. 2022 Dec 1;55:101758. doi: 10.1016/j.eclinm.2022.101758 (PMC9722446; doi:10.1016/j.eclinm.2022.101758)
Supplement: Supplementary Table S1 [file mmc2.docx]

**Supplementary Table**

Table S1: Observed (and Predicted) Mortality by GAP Stage for our cohort (n=999) compared to the literature predicted values
